# Melatonin enhances salt tolerance by promoting *MYB108A*-mediated ethylene biosynthesis in grapevines

**DOI:** 10.1038/s41438-019-0197-4

**Published:** 2019-10-08

**Authors:** Lili Xu, Guangqing Xiang, Qinghua Sun, Yong Ni, Zhongxin Jin, Shiwei Gao, Yuxin Yao

**Affiliations:** 1 State Key Laboratory of Crop Biology, Collaborative Innovation Center of Fruit & Vegetable Quality and Efficient Production, College of Horticulture Science and Engineering, Shandong Agricultural University, Tai-An, Shandong 271018 China; 2 State Key Laboratory of Crop Biology, College of Life Science, Shandong Agricultural University, Tai-An, Shandong 271018 China

**Keywords:** Plant physiology, Plant signalling

## Abstract

The signal molecules melatonin and ethylene play key roles in abiotic stress tolerance. The interplay between melatonin and ethylene in regulating salt tolerance and the underlying molecular mechanism of this interplay remain unclear. Here, we found that both melatonin and 1-aminocyclopropane-1-carboxylic acid (ACC, a precursor of ethylene) enhanced the tolerance of grapevine to NaCl; additionally, ethylene participated in melatonin-induced salt tolerance. Further experiments indicated that exogenous treatment and endogenous induction of melatonin increased the ACC content and ethylene production in grapevine and tobacco plants, respectively. The expression of *MYB108A* and *ACS1*, which function as a transcription factor and a key gene involved in ethylene production, respectively, was strongly induced by melatonin treatment. Additionally, MYB108A directly bound to the promoter of *ACS1* and activated its transcription. *MYB108A* expression promoted ACC synthesis and ethylene production by activating *ACS1* expression in response to melatonin treatment. The suppression of *MYB108A* expression partially limited the effect of melatonin on the induction of ethylene production and reduced melatonin-induced salt tolerance. Collectively, melatonin promotes ethylene biosynthesis and salt tolerance through the regulation of *ACS1* by MYB108A.

## Introduction

Soil salinization is an important environmental problem, and salt has become one of the most commonly encountered abiotic stresses that affects fruit crops, including grapevines, causing nutritional imbalance, ion toxicity, osmotic stress and oxidative damage and severely reducing crop growth, yield and fruit quality. Globally, ~830 million ha of land is affected by salinization, and this area is anticipated to increase in the near future^1^. Grapevines are widely cultivated worldwide and are ranked as sensitive or moderately sensitive to salt stress^2^. It is difficult to obtain highly salt-tolerant grapevine cultivars through traditional breeding methods; in contrast, genetic modification is an effective approach for the creation of new crop varieties with improved characteristics. However, more experimental data are needed to reveal salt tolerance mechanisms that allow crops to optimize their responses to salt. Grapevines and other plants have evolved multiple strategies for protection against salt, one of which is hormone-guided tolerance to salt, including melatonin and ethylene^3,4^. Additionally, plant responses to abiotic stresses are controlled by the interactive hormonal network^5^; plant hormone crosstalk can occur at the level of the regulation of hormone biosynthesis, signal transduction or common target gene expression^6^.

Ethylene is an important signaling molecule mediating numerous important biological processes, including responses to abiotic stresses^7^. The action of ethylene depends on its concentration in cells and the sensitivity of plants to this hormone^8^. Ethylene biosynthesis is primarily regulated by 1-aminocyclopropane-1-carboxylate (ACC) synthase (ACS) and ACC oxidase (ACO) at the transcriptional or posttranslation levels^9^. Salt promotes ethylene production in various species by modulating the activity of ACS and ACO^10,11^. Additionally, the endogenous overproduction of ethylene or exogenous treatment with the ethylene precursor ACC enhances the tolerance of some plants, including *Arabidopsis*^12^ and maize^13^, to salt stress. The inhibition of *ACS* expression reduces salt tolerance in the wild tomato species *Solanum chilense*^14^. In addition to ethylene synthesis, transduction through ethylene responsive factors (ERFs) may also have crucial functions in the plant responses to salt stresses^15^; in addition to the response to salt, ethylene also plays a key role in other stresses, including cold stress in grapevines^16^ and drought stress in soybean^17^. Although substantial progress has been made in identifying the roles of ethylene in abiotic stress responses, more studies are needed to unravel the precise control of ethylene production and signaling in response to abiotic stresses.

In a recent study, we demonstrated that melatonin promoted ethylene biosynthesis and that *MYB108A* and *ACS1* were strongly induced by melatonin treatment during grape berry ripening^18^. Melatonin (N-acetyl-5-methoxytryptamine) is an indoleamine that is synthesized from L-tryptophan metabolism via serotonin. Melatonin is a pleiotropic and highly conserved molecule and is ubiquitous in animals and plants^19^. Melatonin, as a plant regulator, is stress inducible, and both exogenous application and endogenous induction play a key role in enhancing plant tolerance to salt and other abiotic stresses^4,20^. The beneficial role of melatonin in the stress response is broadly attributable to the regulation of the gene expression involved in abiotic stress responses^19–21^. Additionally, there is significant crosstalk between melatonin and other plant growth regulators, including abscisic acid (ABA), jasmonic acid, and salicylic acid^22^; it has been shown that exogenous melatonin application enhances drought priming-induced cold tolerance and drought tolerance by modulating ABA levels in barley and apple, respectively^23,24^. Melatonin also interacts with other signaling pathways; recent studies have reported that mitogen-activated protein kinase pathways (MAPKs) are required for melatonin-mediated defense responses in plants^25^; nitric oxide is required for melatonin-enhanced tolerance against salt stress in rapeseed seedlings^26^.

To date, the molecular pathways associated with crosstalk between melatonin and other signals remain largely unknown. The objectives of this study were to elucidate whether melatonin can regulate salt tolerance via ethylene signals and to reveal the underlying molecular mechanism by which melatonin enhances ethylene biosynthesis via *MYB108A* and *ACS1* in grapevines.

## Results

### Melatonin enhances salt tolerance of ‘Crimson seedless’ grapevines partially through ethylene

**Fig. 1:**
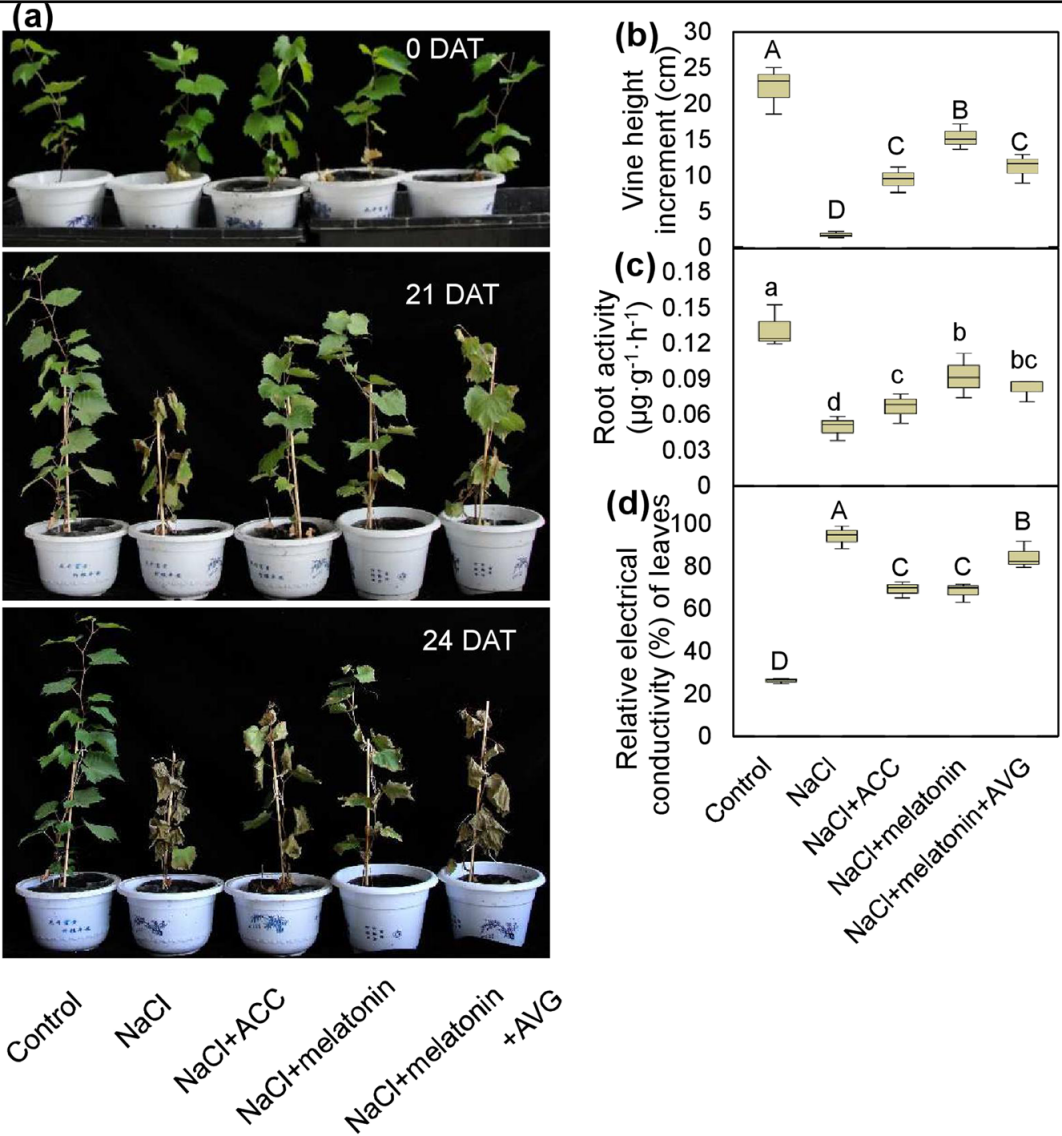
Effects of the addition of ACC, melatonin and melatonin with AVG on the tolerance of ‘Crimson seedless’ grapevines to salt. **a** Phenotypes of grapevines grown in a greenhouse under natural conditions and under various treatments at 0, 21, and 24 DAT. **b**–**d** Vine height increment **b**, root activity **c** and leaf relative electrical conductivity **d** under various treatments at 21 DAT. Vine cuttings were treated with 100 mM NaCl in the presence or absence of 50 µM melatonin, 50 µM ACC and 2 µM AVG. DAT, days after treatment; ACC, 1-aminocyclopropane-1-carboxylic acid; AVG, aminovinylglycine. Values represent the means ± SD of three replicates. Values indicated by the same lowercase and capital letters are not significant at *P* < 0.05 and *P* < 0.01, respectively, on the basis of Duncan’s multiple range test

To investigate whether melatonin and ethylene enhanced tolerance to salt, grapevines were watered with 100 mM NaCl to induce salt stress. After three weeks of NaCl treatment, the grapevines were markedly withered, and the leaves showed necrotic phenotypes; in contrast, the grapevines watered with NaCl plus melatonin or ACC (precursor of ethylene) showed slight marginal necrosis and exhibited less severe phenotypes (Fig. 1a). Additionally, analyses of the vine height increment, relative electrical conductivity and root activity also indicated that the addition of melatonin or ACC alleviated injury by NaCl (Fig. 1b–d). Therefore, ACC and melatonin treatment enhanced the tolerance of vines to NaCl.

Grapevine leaves completely withered when treated with NaCl plus melatonin and aminovinylglycine (AVG, inhibitor of ethylene synthesis) at 24 DAT; in contrast, vine leaves still appeared green, although they exhibited symptoms of marginal necrosis under treatment of NaCl plus melatonin (Fig. 1a). Additionally, the grapevines treated with NaCl plus melatonin and AVG exhibited a lower vine height increment and root activity and a higher electrical conductivity of leaves than those treated with NaCl plus melatonin (Fig. 1b–d). Therefore, AVG decreased the function of melatonin in enhancing vine tolerance to NaCl, indicating that melatonin enhanced vine tolerance to NaCl, partially through ethylene.

### Melatonin increases ethylene production in the roots and leaves of ‘Crimson seedless’ grapevines and tobacco plants

**Fig. 2:**
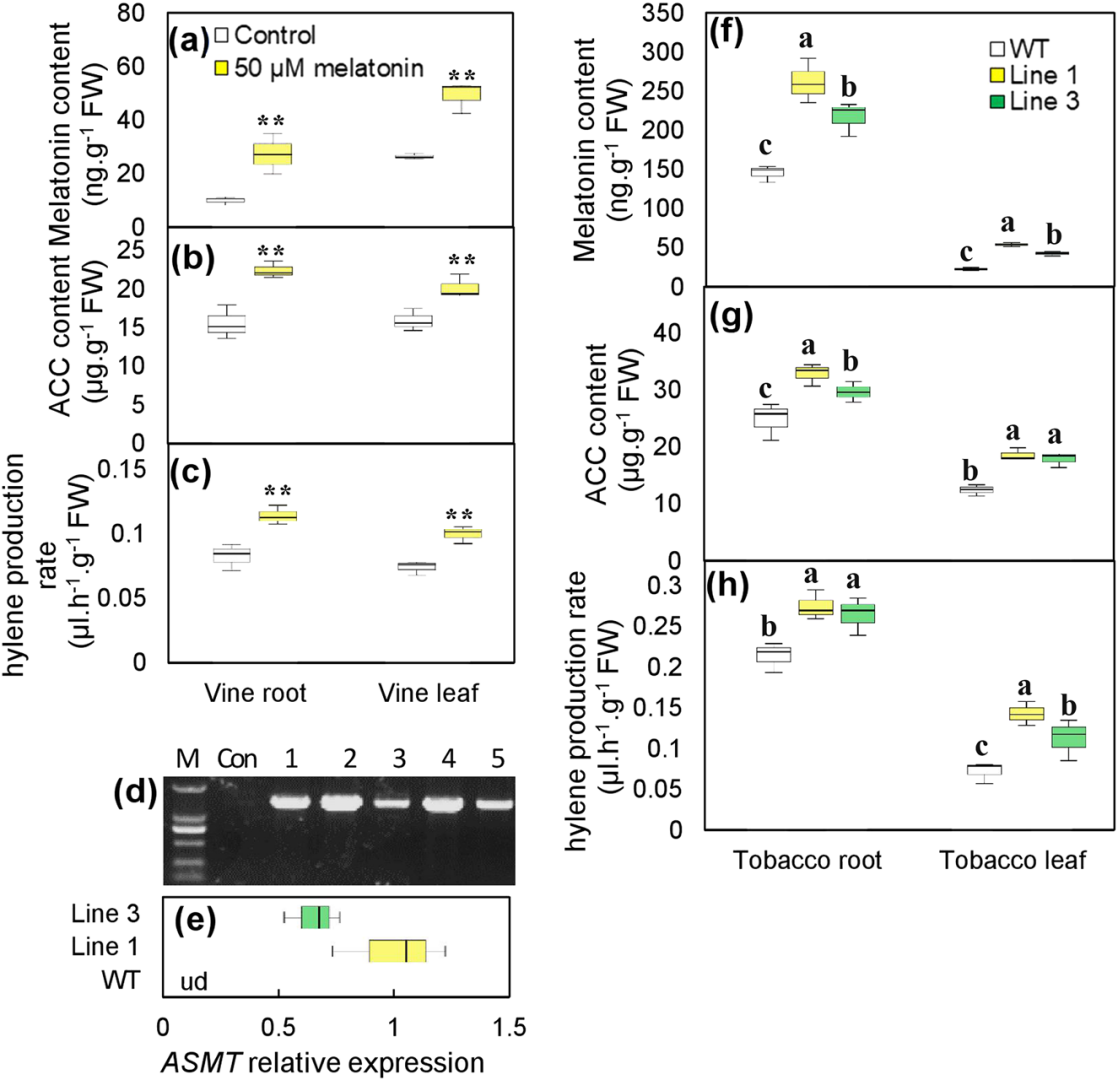
Effects of exogenous application and endogenous induction of melatonin on the ACC content and ethylene production. **a**–**c** Changes in the content of melatonin **a** and ACC **b** and ethylene production rate **c** in ‘Crimson seedless’ vine roots and leaves at 3 days after 50 µM melatonin treatment. **d** PCR identification of *ASMT*-overexpressing tobacco plants using DNA templates and specific primers from *35S* and *ASMT*. **e** *ASMT* expression in tobacco leaves of wild-type (WT) and transgenic lines. **f**–**h** Changes in melatonin **f**, ACC content **g** and ethylene production rate **h** in leaves and roots of WT and transgenic tobacco plants. The numbers represent 5 different transgenic lines of tobacco plants in **d**. ud, undetectable; WT, wild type; M, DNA marker; Con, control; *ASMT*, *acetylserotonin methyltransferase*. Values represent the means ± SD of three replicates. ** highly significant difference, *P* < 0.01. Values indicated by the same lowercase letters are not significant at *P* < 0.05. A one-way analysis of variance (ANOVA) followed by a nonparametric Kruskal–Wallis test was employed

To further investigate the influence of melatonin on ethylene, exogenous melatonin treatment was performed by watering ‘Crimson seedless’ vine roots. The 50 µM melatonin treatment significantly enhanced the melatonin levels in vine roots and leaves (Fig. 2a). As a result, the content of ACC and the ethylene production rate were significantly enhanced in the melatonin-treated roots and leaves compared to the control tissues (Fig. 2b, c). Additionally, grape *acetylserotonin methyltransferase* (*VviASMT*), the final enzyme in the melatonin biosynthesis pathway^27^, was ectopically expressed to increase the biosynthesis of endogenous melatonin in tobacco plants. The transformation was confirmed by PCR detection of the target gene (Fig. 2d), and lines 1 and 3, with different levels of *VviASMT* expression, were selected for further assays (Fig. 2e). *VviASMT* ectopic expression enhanced the melatonin content of roots and leaves in the tobacco transgenic lines (Fig. 2f). The two transgenic plants exhibited significantly higher ACC and ethylene production rates than the control (Fig. 2g, h). Therefore, ethylene production is enhanced by melatonin by both exogenous treatments and the induction its synthesis in planta.

### Melatonin induces the expression of *MYB108A* and *ACS1*, which function as a transcription factor and an ethylene biosynthesis gene, respectively, in ‘Crimson seedless’ grapevine roots and/or calluses

**Fig. 3:**
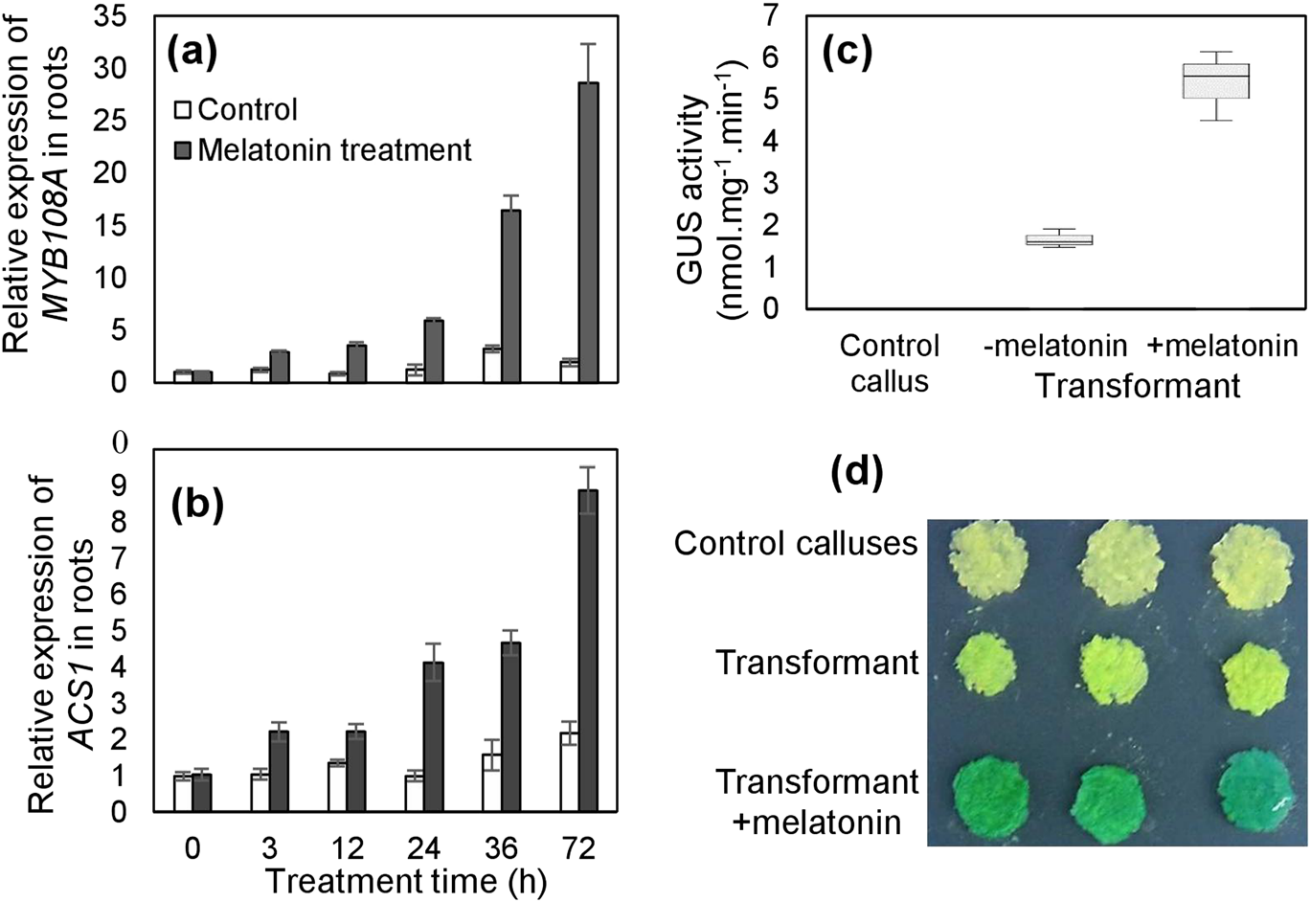
Expression responses of *MYB108A* and *ACS1* determined by qRT-PCR. **a**, **b** and/or GUS activity **c** and staining **d**. qRT-PCRs were performed in the roots of ‘Crimson seedless’ in vitro shoot cultures at different time points **a**, **b**. GUS activity **c** and staining **d** were analyzed using ‘Crimson seedless’ calluses expressing the *MYB108A promoter*::*GUS* (transformant) construct. In this experiment, 50 mM melatonin was employed. In **a**–**c**, values represent the means ± SD of 3 replicates. Photos of GUS staining were taken from the three replicates in **d**

Our previous work demonstrated that *MYB108A* (VIT_205s0077g00500) and *ACS1* (VIT_215s0046g02220), identified in a previous study^28^ and by sequence alignment with its counterpart in *Arabidopsis* (Fig. S1), respectively, were significantly upregulated by melatonin treatment in berries based on RNA-Seq analysis^18^. To further dissect their expression response to melatonin, qRT-PCRs were performed in roots at different time points after melatonin treatment. *MYB108A* and *ACS1* were gradually induced by melatonin and reached a maximum at 72 h, with expression levels that were 13.75- and 3.07-fold higher, respectively, in the treated roots than in the control (Fig. 3a, b). Additionally, histochemical GUS staining and GUS activity were analyzed in the transformed grape calluses expressing the *MYB108A* promoter::GUS (*P*_*MYB108A*_*::GUS*) construct. For transformants with *P*_*MYB108A*_*::GUS*, calluses treated with melatonin produced clearly stronger GUS staining and significantly higher GUS activity than calluses without melatonin treatment (Fig. 3c, d). Therefore, *MYB108A* and *ACS1* were transcriptionally induced by melatonin in vine roots.

**Fig. 4:**
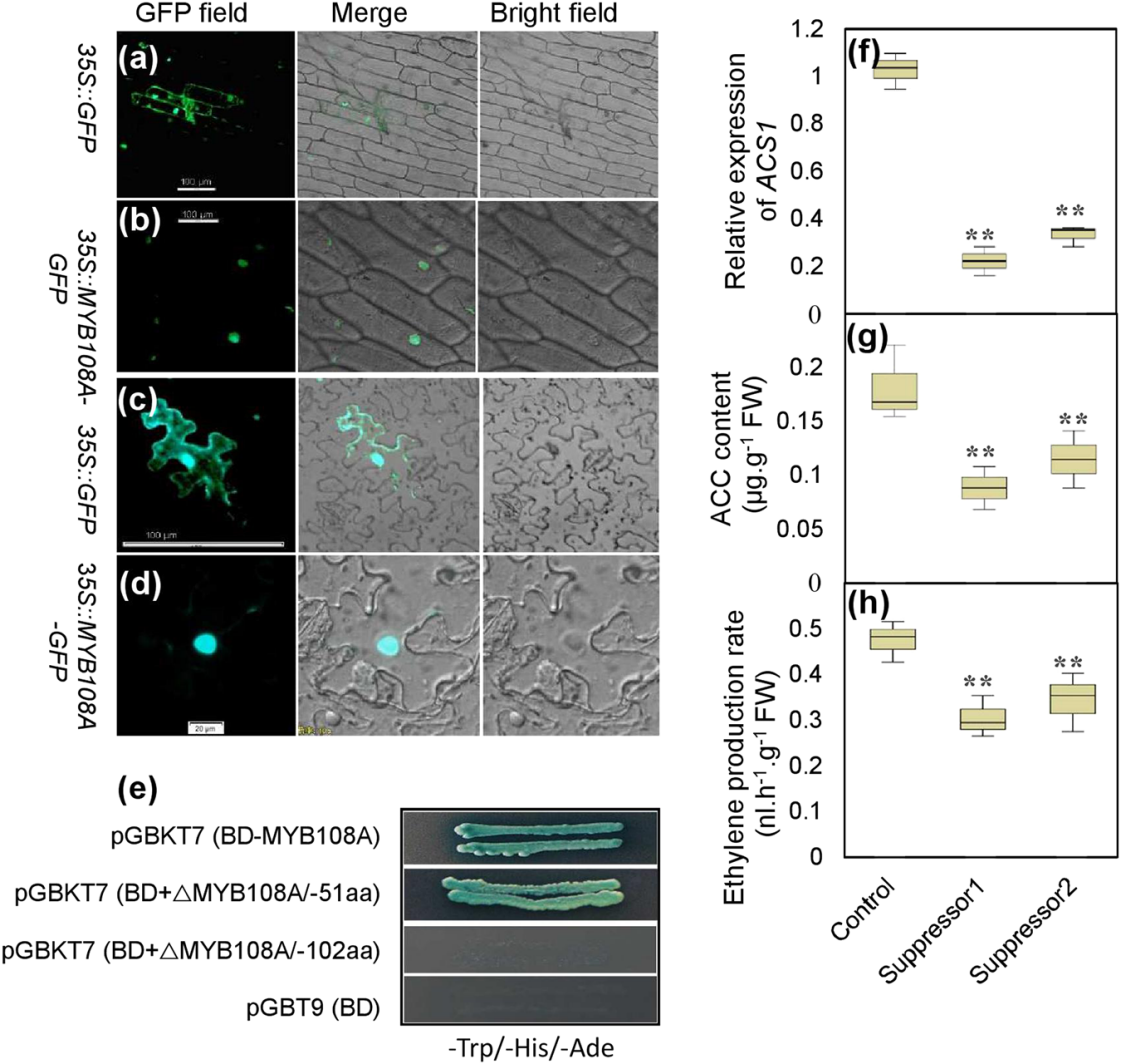
Identification of the properties of MYB108A as a transcription factor. **a**–**e** and the function of ACS1 in the synthesis of ACC and ethylene **f**–**h**. *35S*::*GFP* and *35S*::*MYB108A-GFP* vectors were transiently expressed in onion **a**, **b** and tobacco **c**, **d** epidermis cells. GFP fluorescence, bright field, and overlay merge of bright field and fluorescent illumination are shown **a**–**d**. In **e**, BD indicates the binding domain; △MYB108A/-51aa and △MYB108A/-102aa indicate the deletion of 51 and 102 C-terminal amino acids of the MYB108A protein, respectively. In **f**–**h**, suppressor 1 and suppressor 2 represent *ACS1*-suppressed ‘Crimson seedless’ calluses; values are presented as the means ± SD of 3 replicates; ** highly significant difference on the basis of Duncan’s multiple range test at *P* *<* 0.01

To detect whether MYB108A possessed transcription factor activity, its subcellular location and transcriptional activation properties were determined. The fusion protein MYB108A::GFP was observed in the nucleus of the epidermal cells of onion and tobacco leaves (Fig. 4a, d), while the GFP signal was detected in membrane, cytoplasm and nucleus in the control cells (Fig. 4b, c). Therefore, MYB108A was located in the nucleus. On the other hand, full-length *MYB108A* was subcloned into the pGBKT7 vector containing a GAL4 DNA-binding domain, and this construct was transformed into yeast. Positive X-gal activity was observed in yeast containing pGBKT7-MYB108A but not in yeast containing pGBKT7 on the screening medium (Fig. 4e). These results indicated that MYB108A could activate the expression of the reporter gene in the yeast system. Additionally, the 102 C-terminal amino acids were responsible for transcription activation (Fig. 4e). Taken together, these results indicate that MYB108A is a nuclear transcription factor.

To determine whether ACS1 is a key enzyme controlling ACC biosynthesis, suppressor 1 and suppressor 2 were produced by transforming antisense cDNA fragments matching the *ACS1* 3́-UTR into grape calluses. Compared with the control, the two suppressors had reduced *ACS1* transcript levels, ACC contents and ethylene production rates (Fig. 4f–h); therefore, *ACS1* functions in ACC synthesis.

### MYB108A binds to the promoter of *ACS1* and activates its transcription in yeast, grapevine calluses and tobacco leaves

**Fig. 5:**
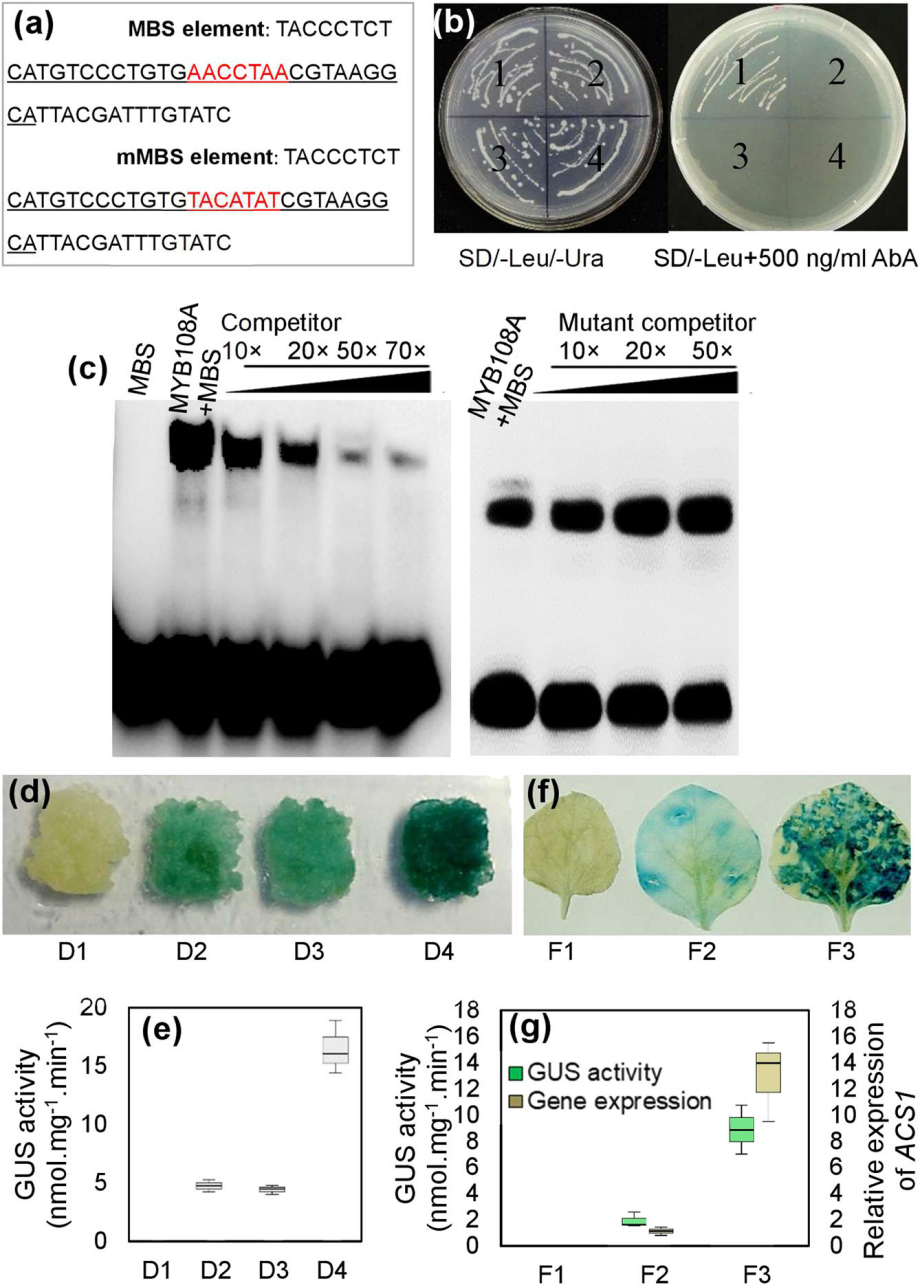
Characterization of transcription activation of *ACS1* by MYB108A. **a** Sequences containing MBS or mMBS elements for yeast one-hybrid assay and underlined sequence for EMSA. **b** Yeast one-hybrid assay using MBS and mMBS as bait. (1) pAbAi-MBS/pGAD-MYB108A; (2) pAbAi-MBS/pGAD7; (3) pAbAi-mMBS/pGAD-MYB108A; (4) pAbAi-mMBS/pGAD7. **c** Interaction of the MYB108A protein with labeled DNA probes for cis-elements of the *ACS1* promoter in EMSA. **d**, **f** Histochemical analysis of the transactivation activity of MYB108A via the binding MBS element. ‘Crimson seedless’ calluses **d** and tobacco leaves **f** were *agro*-infiltrated with different vector constructs: D1, WT calluses; D2, MBS-35S mini-GUS; D3, mMBS-35S mini-GUS and 35S::MYB108A; D4, MBS-35S mini-GUS and 35S::MYB108A; F1, WT leaf; F2, P_ACS1_::ACS1-GUS; F3, P_ACS1_::ACS1-GUS and 35S::MYB108A. **e** GUS activities of grape calluses infiltrated by *Agrobacterium* containing D1-D4 constructs. **g** *ACS1* expression and GUS activities of tobacco leaves infiltrated by *Agrobacterium* containing F1-F3 constructs. Each image is representative of three biological replicates in **b**, **c**, **d**, and **f**. In **e** and **g**, values are presented as the means ± SD of 3 replicates

A yeast one-hybrid assay was performed to determine whether the MYB108A protein was able to bind MBS element (AACCTAA, Fig. S3) in the promoter of *ACS1*. The MBS element or mutant MBS element (mMBS) was inserted into the pAbAi vector, and the corresponding constructs were designated pAbAi-MBS and pAbAi-mMBS, respectively. The complete coding region of *MYB108A* was cloned into the yeast expression vector pGADT7. The resulting pGADT7-MYB108A and pGADT7 constructs were transformed into the yeast strain Y1HGold carrying either pAbAi-MBS or pAbAi-mMBS. All transformed yeast cells were grown on medium without leucine (Leu) and uracil (Ura) to confirm the successful transformation (Fig. 5a). As expected, only the yeast clones containing pAbAi-MBS and pGADT7-MYB108A grew on synthetic dropout medium (SD/-Leu/-Ura) containing 500 µg/L AbA (Fig. 5a), indicating that MYB108A bound to the MBS binding site and activated transcription in the yeast system.

To further verify the direct binding of MYB108A to the MBS-containing recognition site in the *ACS1* promoter, an electrophoretic mobility shift assay (EMSA) was performed with an oligo-probe containing an MBS element using purified recombinant His-MYB108A fusion protein. This indicated that specific DNA-MYB108A protein complexes were detected when the oligo-probe containing the MBS element was used. The formation of these complexes was gradually reduced with the application of increasing amounts of the unlabeled MBS competitor probe with the same sequence (Fig. 5c). In contrast, this competition was not detected when the mutated competitor was used (Fig. 5c). Therefore, the MYB108A protein was able to specifically bind to the MBS element of the *ACS1* promoter.

To investigate whether MYB108A activated gene expression by interacting with the MBS element in plant cells, *Agrobacterium*-mediated transient expression of the GUS reporter gene in grape calluses was performed. The calluses cotransformed with MBS-35S mini-GUS and 35S-MYB108A were bluer in color and showed higher GUS activity than calluses transformed with mMBS-35S mini-GUS and 35S-MYB108A or only MBS-35S mini-GUS (Fig. 5d, e). Therefore, the GUS gene was activated in grape calluses via the interaction between MYB108A and the MBS element. Additionally, the tobacco leaves cotransformed with the P_ACS1_::ACS1-GUS (ACS1-GUS fusion gene driven by the *ACS1* promoter) and 35S::MYB108A, and those transformed with only P_ACS1_::ACS1-GUS were obtained. The results showed that transcripts of *ACS1* and GUS activity were positively regulated by MYB108A (Fig. 5f, g). Therefore, MYB108A acts upstream of *ACS1* to activate its transcript levels.

### Melatonin promotes ethylene production by increasing *MYB108A* expression in ‘Cabernet Sauvignon’ grapevine leaves

**Fig. 6:**
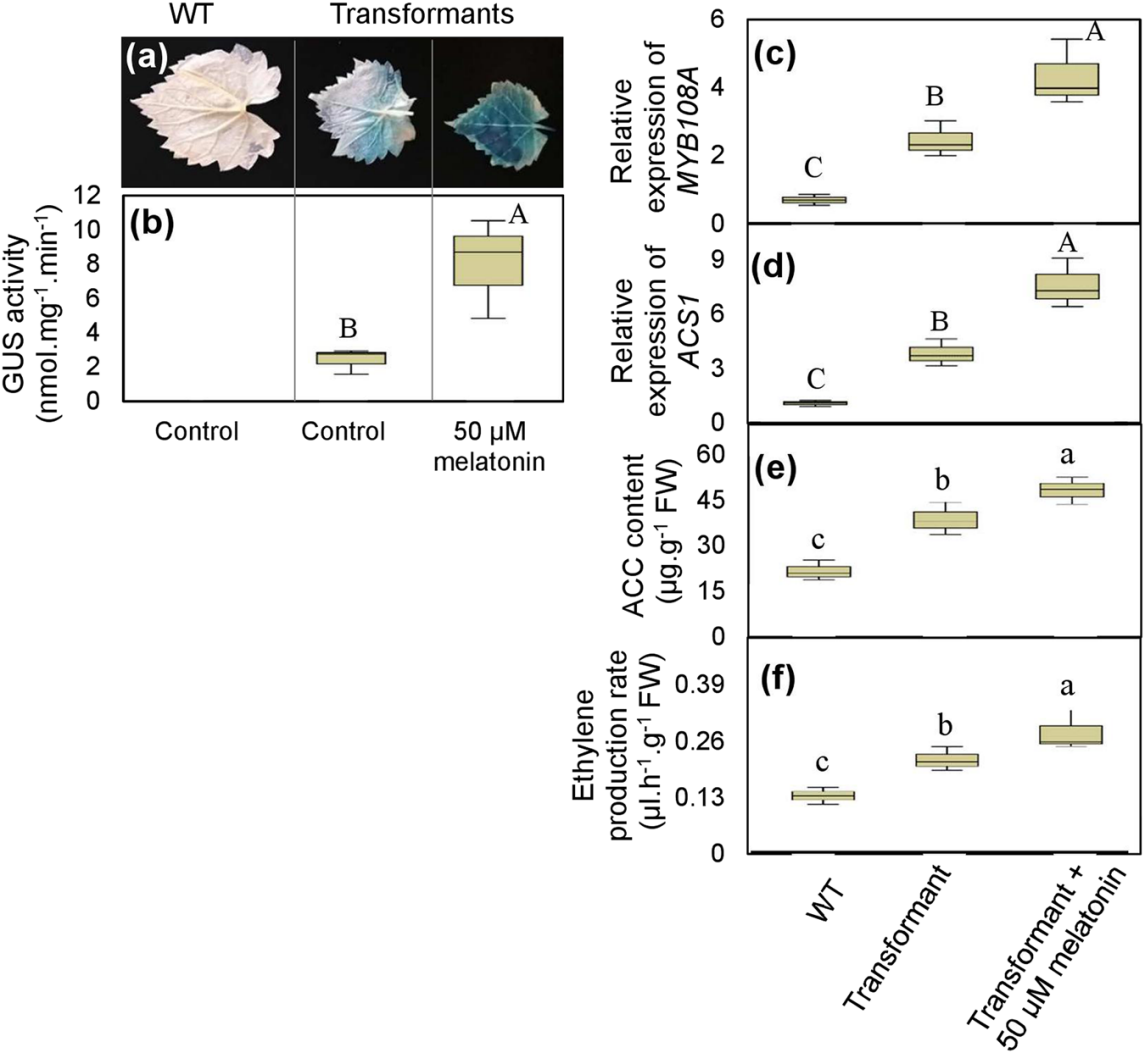
Transient expression of *MYB108A* in response to melatonin and its effects on *ACS1* expression, ACC and ethylene synthesis in in vitro grapevine leaves. Young ‘Cabernet Sauvignon’ vine leaves were infiltrated by *Agrobacterium tumefaciens* containing the construct *P*_*MYB108A*_::*MYB108A*-*GUS*. After 3 days of culture with or without 50 µM melatonin, leaves were used for the determination of GUS staining **a** and activity **b**, relative expression of *MYB108A* **c** and *ACS1* **d**, ACC content **e** and ethylene production rate **f**. WT, wild type; transformant represents the grapevine leaves transiently expressing *P*_*MYB108A*_::*MYB108A*-*GUS*. In **b**–**f**, values are presented as the means ± SD of 3 replicates. Values indicated by the same lowercase and capital letters are not significant at *P* < *0.05* and *P* < 0.01, respectively, on the basis of Duncan’s multiple range test

The fusion gene *MYB108A*-*GUS* driven by the *MYB108A* promoter was transiently expressed in grape leaves to demonstrate whether *MYB108A* can promote ethylene production in response to melatonin. The leaves expressing *proMYB108A*-*GUS* revealed increased GUS staining, GUS activity and *MYB108A* expression compared to the control (Fig. 6a–c). Additionally, the leaves expressing *proMYB108A*-*GUS* exhibited high *ACS1* expression, a high ACC content and a high ethylene production rate (Fig. 6d–f). Therefore, MYB108A activated *ACS1* expression and thereby promoted ACC and ethylene biosynthesis in grapevine leaves.

**Fig. 7:**
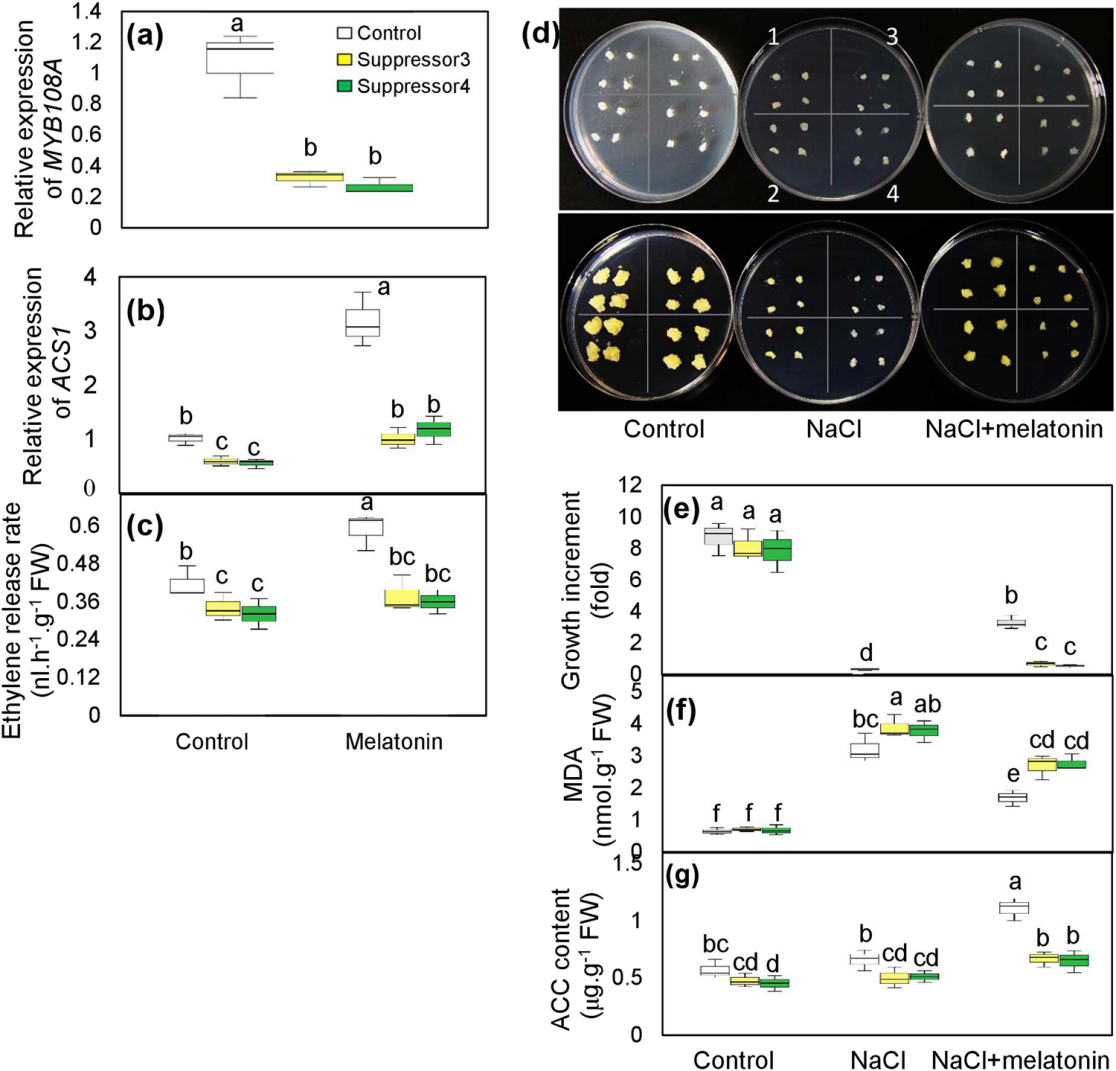
Evaluation of salt tolerance of WT and *MYB108A*-suppressed ‘Crimson seedless’ calluses with or without melatonin. Expression of *MYB108A* **a** and *ACS1* **b** and ethylene release rate **c** were determined using calluses at 21 days after subculture. **d**–**g** Phenotype evaluation **d**, growth increment **e**, MDA content **f** and ACC content **g** of WT and suppressor calluses treated with 100 mM NaCl with or without 50 µM melatonin. In panel d, upper and lower photos were taken at 0 and 21 days after subculture, respectively; WT calluses were indicated by 1 and 2, and suppressor 3 and suppressor 4 were indicated by 3 and 4, respectively, for each culture dish; each image is representative of three biological replicates, and in other panels, different lowercase letters indicate significant difference on the basis of Duncan’s multiple range test at *P* < 0.05

Additionally, the melatonin-treated leaves expressing *proMYB108A*-*GUS* exhibited higher *MYB108A* expression, bluer color and higher GUS activity than the leaves without melatonin treatment (Fig. 6a–c), indicating that *MYB108A* was transcriptionally induced by melatonin. Additionally, the role of *MYB108A* in regulating *ACS1* expression and ACC and ethylene production was strengthened by melatonin treatment (Fig. 6d–f). Moreover, the expression of *ACS1* and ethylene production were significantly reduced in the two groups of *MYB108A*-suppressed grape calluses (suppressor 3 and suppressor 4) compared to the control (Fig. 7a); the melatonin-induced promotion of *ACS1* expression and ethylene production was reduced in the suppressors (Fig. 7b, c). Therefore, melatonin at least partially promoted ethylene production via *MYB108A*.

### *MYB108A* mediates melatonin-induced ethylene production and salt tolerance in ‘Crimson seedless’ calluses

The grape calluses of suppressor 3 and suppressor 4, produced through transforming antisense cDNA fragments of the 3́-UTR of *MYB108A* into grape calluses, were used to evaluate the role of *MYB108A* in melatonin-induced salt tolerance. Under the control conditions, similar growth increments were found for WT and suppressor calluses. However, the growth of WT and suppressor calluses was completely inhibited by 100 mM NaCl (Fig. 7d, e). In contrast, melatonin treatment alleviated the growth inhibition of WT and suppressor calluses caused by NaCl treatment. However, the suppressors still showed much smaller growth increments than the WT calluses under the treatment of NaCl plus melatonin (Fig. 7d, e). Additionally, the suppressors accumulated more MDA than the control under NaCl and NaCl plus melatonin (Fig. 7f), indicating that suppressors were more severely harmed by NaCl with or without melatonin. Taken together, melatonin-induced salt tolerance was negatively affected by the suppression of *MYB108A*. On the other hand, compared to the WT, suppressors possessed a reduced ACC content under all treatment conditions (Fig. 7g). Therefore, the suppression of *MYB108A* expression limited the effect of melatonin on ethylene production.

Taken together, melatonin alleviated salt injury via ethylene signaling, and this process was mediated at least partially by MYB108A.

## Discussion

### *VviMYB108A* participates in the melatonin-mediated signaling pathway in the salt stress response of ‘Crimson seedless’ grapevines

Many studies have demonstrated that melatonin plays a key role in abiotic stress tolerance in multiple species under exogenous application and endogenous induction^4,19^. This study also indicated the role of melatonin in enhancing the salt tolerance of grapevines (Fig. 1). Additionally, rhizospheric treatment with melatonin increased melatonin levels not only in roots but also in leaves (Fig. 2a). Similarly, foliar or rhizospheric treatment with melatonin increased the melatonin levels in untreated roots or leaves, respectively, under both normal and cold-stress conditions^29^. Therefore, local melatonin application might regulate abiotic stress responses in untreated distant parts via long distance transport.

Under abiotic stresses, melatonin not only directly scavenges ROS as a powerful antioxidant but also acts as a signal molecule^4^. The mechanism underlying the melatonin-mediated signaling pathway in plant abiotic stress responses remains largely unknown, but melatonin confers plant abiotic stress tolerance by modulating relevant transcription factors^19^. For example, melatonin induces the transcription of *AtCBFs* and confers enhanced resistance to abiotic stresses; additionally, the transcription factor AtZAT6-activated CBF pathway is essential for melatonin-mediated freezing stress resistance in *Arabidopsis*^30^. Myb4 and AP37 are involved in melatonin-mediated abiotic stresses^31^. Similarly, VviMYB108A mediated melatonin-induced salt tolerance (Fig. 7d–f).

In *Arabidopsis*, R2R3 MYBs are classified into 22 subgroups, and MYB108 and five other MYB proteins (MYB2, MYB62, MYB78, MYB112 and MYB116) belong to subgroup 20^32^. Existing studies indicate that subgroup 20 is involved in abiotic stress responses. For example, *AtMYB112* and *AtMYB108* are significantly induced by salinity in *Arabidopsis* roots and are implicated in both biotic and abiotic stresses^33,34^. *AtMYB2* mediates salt-induced Ca^2+^ signaling and results in salt tolerance in *Arabidopsis* plants^35^. In this study, VviMYB108A, as a member of subgroup 20, also conferred salt tolerance to grape calluses (Fig. S2; Fig. 7d–f). Similarly, the tomato MYB transcription factor SlAIM1, which is also clustered into subgroup 20, enhances resistance to abiotic stress by modulating responses to ABA^36^.

Taken together, *VviMYB108A* functions downstream of melatonin to enhance the salt tolerance of grapevines.

### *VviMYB108A* enhances salt tolerance of ‘Crimson seedless’ grapevines via ethylene

Ethylene is well known as an important positive mediator in plant salt tolerance, such as grapevines (Fig. 1), maize^13^ and tomato^14^. However, in other cases, ethylene levels can adversely affect salt tolerance. For instance, transgenic tobacco plants with poor ethylene biosynthesis exhibit elevated salt tolerance, and the treatment of rice plants with ethylene confers salt hypersensitivity^15,37^. Similarly, the role of ethylene in cold tolerance varies with plant species. For example, ethylene enhances cold tolerance in grapevine^16^; in contrast, ethylene negatively influences the cold tolerance of *Medicago truncatula*^38^. Therefore, the role of ethylene in abiotic stress tolerance is influenced by the plant species and even the developmental stage^39^; additionally, fine-tuning ethylene biosynthesis may be essential to abiotic stress tolerance in plants^7^; moreover, homeostasis between ethylene and its receptors may affect the function of ethylene in salt stress^40^.

As a rate-limiting enzyme, ACS is the major target for the regulation of ethylene production under stress conditions^40^. *VviACS1* has been shown to respond to salt and to be responsible for ethylene biosynthesis (Fig. 1b; Fig. 4f–h). Similarly, *ACS1a* and *ACS1* are the major genes responsive to salt stress in maize and tobacco, respectively^13,41^. In addition to *ACS1*, several other *ACSs*, such as *ACS2* and *ACS7*, have also been reported to be induced by salt stress in *Arabidopsis*^10^. However, the *Arabidopsis acs7* mutant, which displays reduced ethylene production, exhibits increased salt tolerance at the seed germination stage^42^, suggesting the complex regulation of ethylene synthesis catalyzed by ACSs in response to salt stress. In cotton, a series of ACSs are upregulated under both short- and long-term salinity conditions^43^. In this study, compared to the substantial decline in *VvACS1* expression in the suppressors, a relatively small decrease in the ACC content and ethylene production rate was detected (Fig. 4f–h), suggesting the roles of other ACS isoforms in controlling ethylene synthesis in grapevines. Additionally, among the promoters of 21 *ACS* genes in soybean, all contain at least one MYB binding cis-acting element^20^, suggesting that their expression is regulated by MYBs. It was also verified that *MYB1* overexpression increases ethylene production and upregulates the expression of ethylene synthetic genes, including *ACS1*, *ACS3a*, *ACS4* and *ACS6* in apple; in contrast, the results were obtained in the *MYB1*-suppressed tissues^43^, suggesting the transcriptional regulation of MYB1 on *ACS* expression. Additionally, *VviMYB108A* is coexpressed with *VviERG1*, *VviERF113* and *VviERF114*, which are involved in ethylene signaling^28^. In this study, *VviACS1* was identified to be directly transcriptionally activated by VviMYB108A (Fig. 5). Taken together, VviMYB108A promoted ethylene production by increasing *VviACS1* expression and thereby enhanced the salt tolerance of grapevines.

### The regulatory effect of melatonin on ethylene synthesis may be related to complex hormone signal crosstalk

In grapevines, melatonin enhanced the salt tolerance of vines and promoted berry ripening, which was related to ethylene production (Fig. 1)^44^. Additionally, *VviACS1* was shown to be a target gene induced by melatonin (Fig. 5). In contrast, melatonin treatment promotes tomato postharvest ripening, and the ethylene production level correlates well with *ACS4* expression^45^. However, melatonin treatment reduced ethylene production and resulted in delayed postharvest banana ripening and pear fruit senescence through the regulation of the expression of *ACO1* and *ACS1*^46,47^. In particular, melatonin treatment inhibits ethylene production in banana leaves, but combined treatments of melatonin and *Fusarium wilt* induce ethylene levels^48^. Therefore, it has been suggested that the function of melatonin in regulating ethylene biosynthesis may be indirect, and its positive or negative influence on ethylene production may be affected by other factors, possibly including additional signal molecules.

Recent studies have indicated that melatonin regulates sugar accumulation and metabolism in apple and tobacco plants^49,50^; changes in sugar status evoke the generation of sugar signals that are integrated with multiple hormone signaling pathways^51^; therefore, melatonin might act through interactions with sugar and hormone signaling pathways. Additionally, cumulative studies have shown that melatonin is an important modulator of gene expression related to plant hormones, such as IAA, ABA, gibberellins and ethylene^24^, and this modulation might play a key role in melatonin-mediated tolerance to abiotic stress. For example, melatonin promotes seed germination under high salt by regulating the ABA and GA4 interaction in cucumber^52^ and results in higher ABA concentrations in drought-primed plants when exposed to cold stress^53^. However, ethylene biosynthesis is controlled via crosstalk with other hormones. For example, ethylene biosynthesis is tightly controlled by cytokinins^54^. In particular, our previous work demonstrated that melatonin treatment promoted ethylene production via ABA^44^. In this study, *VviMYB108A* promoted ethylene synthesis in response to melatonin (Fig. 6); additionally, *AtMYB108* was also induced by ABA in *Arabidopsis* roots^55^. Therefore, it is suggested that melatonin possibly upregulates *VviMYB108A* through ABA.

Taken together, these results suggest that melatonin may regulate ethylene biosynthesis via complex crosstalk with other signal molecules and that the signaling molecules involved may at least partially determine the effect of melatonin on ethylene in a positive or negative manner. This hypothesis also explains why melatonin induced the expression of different *ACS* genes in the above-mentioned species, and ACC and ethylene syntheses are not directly proportional to melatonin production (Fig. 2).

## Conclusions

Melatonin and ethylene enhanced the salt tolerance of grapevines under the conditions applied in this study. Ethylene production was enhanced by melatonin, and ethylene participated in melatonin-induced salt tolerance. Further analysis revealed that *MYB108A* expression was strongly induced by melatonin, and MYB108A could directly bind to the promoter of *ACS1*, activating its expression and promoting ethylene synthesis; MYB108A played a key role in the effect of melatonin on ethylene synthesis. Taken together, a pathway for melatonin-induced salt tolerance was revealed, i.e., “melatonin–*MYB108A*–*ACS1*–ethylene synthesis–salt tolerance”.

## Materials and methods

### Plant materials, growth conditions and experimental treatments

‘Crimson seedless’ (*Vitis vinifera*) cuttings were used for salt tolerance assays. They were planted in 15-cm-diameter plastic pots filled with a 2:1 (v/v) mixture of soil:vermiculite in a greenhouse under normal conditions. For the salt assay, vine cuttings were watered every 3 days with 100 mM NaCl in the presence or absence of 50 µM melatonin, 50 µM ACC and 2 µM AVG.

‘Crimson seedless’ in vitro shoot cultures were used for the determination of melatonin, ACC, ethylene production rate and gene expression. Five-week-old uniform vines were treated with Hoagland’s nutrient solution (control) and 50 μM melatonin in glass bottles with a 10-cm height and 6-cm diameter. Each glass bottle was provided with sufficient oxygen with an oxygen machine (SenSen Group, China). The vines were grown in a controlled-environment growth cabinet with a temperature of 25 °C, a 14-h photoperiod and a light intensity of 600 μmol/m^2^/s.

‘Crimson seedless’ grape calluses were used for gene transformation and the salt tolerance assay. The callus was subcultured on MS medium supplemented with 0.59 g/L 2-(N-Morpholino) ethanesulfonic acid, 10 mg/L picloram, and 2.2 mg/L thidiazuron at 25 °C under dark conditions. For the salt assay, MS medium was supplemented with 100 mM NaCl alone or combined with 50 µM melatonin.

The young leaves of ‘Cabernet Sauvignon’ grapevines cultivated in the field were used for transient transformation. The leaves of *Nicotiana benthamiana* seedlings were used for the transformation of *AMST*. They were planted in 10-cm-dimeter plastic pots with culture stroma and grown under a 16-h light/8-h dark photoperiod at approximately 600 µmol/m^2^/s and 28 °C.

### Determination of the root activity, relative electrical conductivity and malondialdehyde (MDA) content

Root activity was measured using the triphenyl tetrazolium chloride (TTC) method^56^. The relative electric conductivity was measured and calculated as described by Zhou and Leul^57^. MDA was determined using the thiobarbituric acid reactive substances assay, as previously reported^58^.

### Determinations of melatonin, ACC and ethylene production rate

Melatonin was extracted according to our previous study^18^. The samples were separated on a Waters Acquity UHPLC system (Milford, MA, USA) equipped with a BEH C_18_ column (Waters, 2.1 mm internal diameter ×50 mm length, and 1.7 µm particle size). Mass spectrometry (MS) analyses were performed using a QTof-Micro mass spectrometer. The detection parameters and conditions of the ultra-high-performance liquid chromatography (UHPLC)-MS analysis were set according to our previous study^26^.

The ethylene production rate was measured as described in a previous study^18^. ACC extraction and determination were performed according to the method described by Tucker et al.^59^.

### RNA extraction and quantitative RT-PCR

Total RNA was isolated using RNA plant Plus Reagent (Tiangen, Beijing, China). qRT-PCR was performed using the Ultra SYBR Mixture (SYBR Green I) (CWBIO, Beijing, China) in an ABI7500 qRT-PCR instrument (ABI, MA, USA) according to the manufacturer’s instructions. *VvUBI* was used as the internal reference. The specific primers of the amplified genes are listed in Supplementary Table S1.

### Subcellular localization of the MYB108A protein

The *MYB108A* open reading frame was isolated and cloned into the binary vector pROKII-GFP downstream of the *35S* promoter. The resultant construct *35S::MYB108A-GFP* was introduced into *Agrobacterium tumefaciens* GV3101 and transformed into onion epidermal cells^60^ and leaves from 5-week-old *N. benthamiana* seedlings^61^. After 2-3 days of incubation, the subcellular localization of GFP was monitored with an epifluorescence microscope (Olympus BX53F, Tokyo, Japan).

### Transformation of *MYB108A*, *ACS1* and *ASMT* into grape calluses and/or tobacco plants

The complete coding regions of *MYB108A* and *ASMT*, used for sense overexpression, were isolated from ‘Crimson seedless’ roots and cloned into the pRI101-AN (Takara, Dalian, China) vector downstream of the *35S* promoter. The 3́-UTR sequences of *MYB108A* and *ACS1* were cloned into the same vector for antisense suppression. The resultant constructs were introduced into *Agrobacterium* strain LBA4404 and transformed into grape callus, as described by Li et al.^62^ with some modifications, and into tobacco leaves, as reported by Wang et al.^63^. For grape callus transformation, grape calluses were immersed in an *Agrobacterium* suspension for 20 min, blotted dry on sterile filter paper and transferred to solid MS medium with 100 µM acetosyringone. After two days of coculture in darkness at 25 °C, the calluses were transferred to the screening medium supplemented with 100 mg/L kanamycin and 300 mg/L cefalexin. Five weeks later, most of the calluses had died, and the obtained calluses were subcultured on screening medium at 4-week intervals. The *ACS1*- and *MYB108A*-suppressed grape calluses from two independent *Agrobacterium*-mediated transformations were obtained and designated as suppressors 1 and 2 and suppressors 3 and 4, respectively.

### Binding assays of MYB108A to the MBS element using a yeast one-hybrid system and EMSA

For the yeast one-hybrid assay, the MBS element was synthesized and inserted into the pAbAi vector. The ORF of *MYB108A* was amplified and fused in-frame with the GAL4 activation domain of the pGADT7 vector. The mutant MBS (mMBS) was used as a negative control. The resultant plasmid was introduced into the yeast strain Y1HGold. The detailed procedure was performed according to the user manual for the Matchmaker Gold Yeast One-Hybrid Library Screening System (Clontech, Mountain View, CA, USA).

For EMSA, the *MYB108A* ORF was cloned into the expression vector pEASY-E1 (TransGenBiotech, Beijing, China). The MYB108A-His recombinant protein was expressed in *Escherichia coli* strain BL21 and purified using HIS-tag BeaverBeads™ Nickel (Beaver, BioBAY, China). The *ACS1* promoter probe containing an MBS element was synthesized and labeled with biotin (Sangon, Shanghai, China). Unlabeled competitor probes were generated from the dimerized oligos of the *ACS1* promoter regions containing the MBS element. EMSA was performed as described in the instruction manual included with the EMSA Kit (Thermo Fisher Scientific, MA, USA).

### Assay of the *MYB108A* promoter activity

The 1872-bp promoter of *MYB108A*, used for expression response, was isolated and fused to pRI101-GUS via replacing its *35S* promoter (*P*_*MYB108A*_::*GUS*). The resultant construct was introduced into *Agrobacterium* strain LBA4404 and transformed into grape callus, as described above. GUS histochemical staining was performed according to the methods of Jefferson et al.^64^. The GUS activity was calculated as nmol of 4-methylumbelliferone (4-MU) per mg protein per minute.

### Transient transformation of *MYB108A* into different tissues

The complete coding region of *MYB108A* was inserted upstream of GUS in the construct *P*_*MYB108A*_::*GUS*, and the resultant construct was designated *P*_*MYB108A*_::*MYB108A-GUS*. The constructed plasmids were introduced into *Agrobacterium* strain GV3101. The *Agrobacterium*-mediated transient transformation of ‘Cabernet Sauvignon’ leaves was performed, as previously described^65^.

Transient cotransformation was used to determine whether MYB108A could bind to the MBS element and activate the expression of downstream genes. The synthesized sequences containing MBS and mMBS were fused upstream of the 35S minimal promoter of pRI101-GUS (Takara, Dalian, China) to generate the MBS and mMBS mini-GUS plasmids. The promoter sequence of *ACS1*, 1500 bp upstream of ATG, was used to replace the 35S promoter of pRI101-GUS, and the *ACS1* ORF was inserted upstream of GUS, generating the *P*_*ACS1*_::*ACS1-GUS* plasmid. The above two plasmids and the plasmid of *35S*::*MYB108A*, constructed above for the sense overexpression of *MYB108A*, were introduced into *Agrobacterium* strain GV3101. The *Agrobacterium*-mediated transient transformation of tobacco leaves was performed according to Yang et al.^66^. The *Agrobacterium*-mediated transient transformation of grape calluses was performed according to the same procedure of infiltration and coculture in genetic transformation of calluses mentioned above.

### Transactivation property assay of MYB108A

The yeast two-hybrid system was employed to determine the transcription activation property of MYB108A as a transcription factor. The complete *MYB108A*-coding sequence, *△MYB108A/-51aa* or *△MYB108A/-102aa* was isolated and cloned into pGBKT7 to generate an in-frame fusion with the GAL4 DNA-binding domain. The plasmid pGBKT7-MYB108A, pGBKT7-△MYB108A/-51aa or pGBKT7-△MYB108A/-102aa was transformed into yeast. SD media supplemented with -Trp/-His/-Ade and 5-bromo-4-chloro-3-indolyl-d-galactopyranoside acid (x-α-gal) was used to test for possible transcription activation.

### Statistical Analysis

All statistical analyses were performed by SPSS (V19.0) software. A one-way analysis of variance (ANOVA) followed by Duncan’s multiple range test and/or nonparametric Kruskal–Wallis test were employed, and standard deviation (SD) was calculated from three replicates.

## Supplementary information

Supplementary data

## Data Availability

Data supporting the results can be found in this paper.
