# Supplementary material for: Melatonin enhances salt tolerance by promoting MYB108A-mediated ethylene biosynthesis in grapevines
Source: Hortic Res. 2019 Oct 8;6:114. doi: 10.1038/s41438-019-0197-4 (PMC6804660; doi:10.1038/s41438-019-0197-4)
Supplement: Supplementary file 1 — Supplementary data [file 41438_2019_197_MOESM1_ESM.docx]

**Supplementary data**

**Table S1.** Primers used in this study

| **Names of primer pairs**  **or genes** | **Forward primers (5ʹ-3ʹ)** | **Reverse primers (5ʹ-3ʹ)** | **Notes** |
| --- | --- | --- | --- |
| *VvACS1* | TGTGATGTCCCCTCATTCCC | CGCATCTAACTGCCAACCAA | qRT-PCR |
| *VvMYB108A* | ACGCAGTTCACCAACATCTG | AACCTCCAATCCAGTTATCTAC | qRT-PCR |
| *VvASMT* | CCGAATAAGACACTGTGCG | TGGAAAGCAAGAAGGGTG | qRT-PCR |
| *VvUBI* | GTGGTATTATTGAGCCATCCTT | AACCTCCAATCCAGTTATCTAC | qRT-PCR |
| *VvMYB108A-01* | GGATCCATGGATGTTAATGGTAGAGGTGGTA | CTCGAGGATGTTGGTGAACTGCGTC | Subcellular localization |
| *VvMYB108A-02* | GTCGACATGGATGTTAATGGTAGAGGTG | GGATCCTCAGATGTTGGTGAACTGCGTC | Overexpression |
| *VvASMT-01* | GAGCTCATGGATTTGGCAAATGGTGAGAG | GGATCCAGGATAAACCTCAATAAGAGAC | Overexpression |
| *VvMYB108A-03* | GGATCCATGGATGTTAATGGTAGAGGTGGT | GTCGACTGTTCTTCGAGAGTGATGTTTCC | Antisense suppression |
| *VvACS1-01* | GGATCCATTAGCCAGTTCAAAGATGTCGC | GTCGACCTGTGTTCTCCATCTCAAGTCCC | Antisense suppression |
| *VvMYB108A-04* | AAGCTTCTCAACATCATTTACATCCTA | GTCGACTCCGAACAAAGTCTCTGATTGATT | Amplifying the promoter of *VvMYB108A* |
| *VvACS-02* | AAGCTTAAAAAAAGGTGAAAGAACGG | GTCGACTAGCAAAGCAATGAGAGTGG | Amplifying the promoter of *VvMYB108A* |
| *VvMYB108A-05* | GGATCCCAGAGACTTTGTTCGGAAT | CTCGAGATCAGAGATGTTGGTGAAC | Yeast one-hybrid experiment |
| *VvMYB108A-06* | ATGGATGTTAATGGTAGAGGTGGTA | TCAGATGTTGGTGAACTGCGTC | EMSA |
| *VvMYB108A-07* | CATATGATGGATGTTAATGGTAGAGGTGGT | GGATCCTCAGATGTTGGTGAACTGCGTC | Transactivation assay |
| *MYB108A△51* | CATATGATGGATGTTAATGGTAGAGGT | GGATCCCAAATTGTCCGCCGTGGCCGTGTC | Transactivation assay |
| *MYB108A△112* | CATATGATGGATGTTAATGGTAGAGGT | GGATCCGGAAATCCAATCCTTGGTTGAAGT | Transactivation assay |

Restriction Enzyme cutting sites are underlined





VIT_215s0046g02220

**Fig. S1** **Phylogenetic tree of VIT_215s0046g02220 and *Arabidopsis* ACSs**.

Tree was constructed by Neighbor-Joining method and 1000 bootstraps using Mega 4 software.

AAGAGAGAAAAAAAAAAAGGTGAAAGAACGGTCTGGACTAATTTTTCTTTCCATGTATAGAAATCTCTCA -1547

CATCTTCATATGGTTTTGTTATTGAATCTTTTATTAACATTGAAGTGTTGGTTTTATATTCAAGAATCAA -1471

ATTGAATGATTGGTTCTATACTTATAAATCGATCACCCTCATAGTCCCGTGGTTGGACATTTGACCCATA -1401

AGCATGACTGTGATACTAATTTGTAATTATTTTCATTCCTTTCATATTCTTTAAAATCATTTGATGGAAT -1331

AACAATTGCGATAATCAGTTCTTTAAATCAAGTCAATCCTTTTACAAGTTTACACCCTTGGAATTGTACC -1261

CTCCATATAGTCTTGTTATCAAATCTTTTATTAACATTGAAGTGTTGGTTTAATATATATTCAAGAATTG -1191

AATAATTCATTCTATACACGAAGATCGATCACCCCTATAATCACATTGTTGGATAATTTGACTCATAAAC -1121

ATAACTTTTATACCAATTTGAAATTGTCTTCATTTATTTCACATTCTTTAAAATTATTTGGTGGAATAAT -1051

GATCGTGATAATCAGTCCACCAAAATAAGTCAATCCTGCTACAAGTCTACGACCTAGAATTGTCTCAATC -981

CTCTACTAACATTTTTTCCAATTTACACTACAATAATTCCCAAAAAAATGAGAATTGGGTCCATATTAAT -911

TTTAGATTTAACAGATGGAAACCTCTAATACCCACTGCGTAGTCTACTTGCCTGCTCGCTTTTTGGTACA -841

AAAGGAAACCATTGAGAACTTATTGGCTTAAAGATGGCCATCATTATATATTGAAAATCAAATGTAGGTT -771

GCGACATCTCACATCCGCCAACTAAAAGTGAAGTCGGTACCATGTACACTTATTTTTTTGATGACACTAC -701

CCCATTTGCTTTTGCCCCACCACAACCCTAACATTTTCCATAACCTGTCGGCCGATCTCCGTGTGCTGGC -631

CTTGGCTAACCGTCCTCCGACGGGGGTCCTCCCGACAATGAATATTTGCCGTGAACATTTTGTTATACTT -561

TATCTTATTTCTTCGTATTTTGGTCTGATATTTGAACTTTGATGGATACGAGTTTATATTGTTGTCTCCG -491

ATTAAATTTGTGTCTAGTGTTTTGTGTGACTCTTACCCGGACCAAAAGACAAAGGCAAAAAAGTCAAAAG -421

AGCCGCCCAATTTCAAACCTTGTGAATCTCCTGATTCTTCGTGGGAACTTCTTCCTCGTATTTTCCCACT -351

AAAAAATTTTCCCTCTGCAAGAGCCTAACAAATTAGATTCAACCTGGGAAAAAAAAATAGAATTCAGTAG -281

TCTTTTGGTTCTTACCCTCTCATGTCCCTGTGAACCTAACGTAAGGCATTACGATTTGTATCCCCACGTC -211

ATATGGTCACTTCCCATTTTTCTCATTTTCTGGGGGATTTGGCTTACCTTCTTACTTTGAAAATTTTCCA -141

TTTCCCCATTTTTGGTCCGTGTTCAACTGTACAGAGGTCTATAAAATTCTCTTGCATTCTCACATATTCC -71

CCCACAGCTATCGGCATTTCCCACTCTCATTGCTTTGCTAGCTACATATCCAGAGATACACTTAGAAACC -1

**ATG**

**Fig. S2 *In silico* analysis of MYB binding elements in the promoter of *VvACS1*.**

MYB Binding element, highlighted with yellow color, was analyzed through PlantCARE Search Tool (http://bioinformatics.psb.ugent.be/webtools/plantcare/html/).
